# Patterns Exploration on Patterns of Empirical Herbal Formula of Chinese Medicine by Association Rules

**DOI:** 10.1155/2015/148948

**Published:** 2015-10-01

**Authors:** Li Huang, Jiamin Yuan, Zhimin Yang, Fuping Xu, Chunhua Huang

**Affiliations:** ^1^The Second Affiliated Hospital of Guangzhou University of Chinese Medicine, 111 Dade Road, Guangzhou 510120, China; ^2^Neurology Department, Jiangxi Provincial Hospital of Chinese Medicine, Nanchang 330006, China

## Abstract

*Background*. In this study, we use association rules to explore the latent rules and patterns of prescribing and adjusting the ingredients of herbal decoctions based on empirical herbal formula of Chinese Medicine (CM).* Materials and Methods*. The consideration and development of CM prescriptions based on the knowledge of CM doctors are analyzed. The study contained three stages. The first stage is to identify the chief symptoms to a specific empirical herbal formula, which can serve as the key indication for herb addition and cancellation. The second stage is to conduct a case study on the empirical CM herbal formula for insomnia. Doctors will add extra ingredients or cancel some of them by CM syndrome diagnosis. The last stage of the study is to divide the observed cases into the effective group and ineffective group based on the assessed clinical effect by doctors. The patterns during the diagnosis and treatment are selected by the applied algorithm and the relations between clinical symptoms or indications and herb choosing principles will be selected by the association rules algorithm.* Results.* Totally 40 patients were observed in this study: 28 patients were considered effective after treatment and the remaining 12 were ineffective. 206 patterns related to clinical indications of Chinese Medicine were checked and screened with each observed case. In the analysis of the effective group, we used the algorithm of association rules to select combinations between 28 herbal adjustment strategies of the empirical herbal formula and the 190 patterns of individual clinical manifestations. During this stage, 11 common patterns were eliminated and 5 major symptoms for insomnia remained. 12 association rules were identified which included 5 herbal adjustment strategies.* Conclusion.* The association rules method is an effective algorithm to explore the latent relations between clinical indications and herbal adjustment strategies for the study on empirical herbal formulas.

## 1. Background

Empirical herbal formula is a relatively stable combination of herbs under the theoretical framework of Chinese Medicine. The development of empirical herbal formula undergoes long-term history of clinical practice and it is on the basis of the summarized experience of a variety of senior Traditional Chinese Medicine (TCM) doctors. Nowadays, the development of TCM clinical guidelines also stresses the use of empirical herbal formulas which are considered as valuable heritage of TCM. In order to promote the clinical use of these formulas, it is needed to interpret them with clear and accurate descriptions with rigorous scientific language. However, the previous research on empirical herbal formulas of TCM is subjective in the fact that they are simply summaries by the TCM doctors or their apprentices. This kind of subjective study methods causes two major demerits: at first, the adjustment of herbal components in the empirical formula is guided by the experience and knowledge of the doctors. Therefore, the subjective summary for the potential knowledge and implicit rules is not sufficient and complete [1]. And furthermore, it usually needs longer time to implement this kind of research. On the other hand, the results of the summarization by people with different knowledge background and preference are usually inconsistent; thus the study reliability and stability cannot be confirmed. The application of empirical herbal formulas can be improved and promoted to other doctors only after they are to be studied and explored by objective and reliable methods. And then we will be able to inherit the valuable clinical experience of senior TCM doctors.

In order to carry out this task, we imitated a study on the empirical herbal formula si-ni and gui-gang-long-mu Tang by Professor Zhimin Yang, which is a herbal decoction originated from classic TCM prescriptions to treat insomnia. The association rules based on machine learning are used to explore the patterns of herbal combination strategy in real practice of Professor Zhimin Yang. The method of association rules is a well-known informatics approach to explore the latent correlation among a variety of factors [2]. The technology of informatics is being introduced to TCM, and a variety of studies were implemented previously. For example, Zhang et al. introduced a machine learning model to assess the efficacy of acupuncture by establishing a high quality training set [3] with the prediction accuracy over 80%. Liang et al. introduce a* k* nearest neighbor (*k*NN) algorithm to analyze different efficacy parameters of acupuncture for neck pain, and the research indicates high reliability for analyzing multidimensional outcomes [4].

In this study, an algorithm based on association rules is developed to explore the latent correlation among herbal components in empirical formulas and clinical indication and symptoms detected by examination. It is a good study example and attempt to use machine learning to explore the herb prescribing patterns of TCM doctors.

## 2. Materials and Methods

### 2.1. Ethics Statement

The research protocol of this study is reviewed and approved by the Ethics Committee of Guangdong Provincial Hospital of Chinese Medicine. All research activities comply with the principles of Declaration of Helsinki (64th WMA General Assembly, Fortaleza, Brazil, October 2013). The personal information of all included patients is concealed against any exposure to the public. No information or clinical photographs relating to individual patients are included in this paper.

### 2.2. Study Rationale

**Figure 1:**
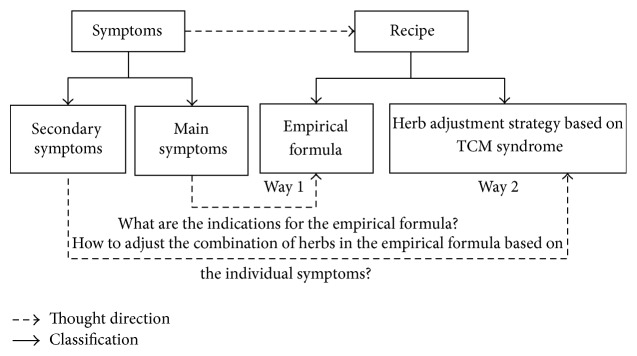
Procedure of developing and selecting an empirical formula.

Because of the theoretical and related clinical practice discrepancy of Western medicine and TCM, the TCM doctors actually undergo a different thinking procedure in their clinical visits by patients. Therefore, before we begin to develop the herbal prescriptions, we need to understand the development procedure of the formula or the mechanism of selecting empirical formula for a specific problem. An example of the procedure is illustrated in Figure 1.

As shown in Figure 1, TCM doctors view all organic systems of the human body as a whole. A TCM syndrome diagnostic system will be applied under the framework of TCM knowledge. In TCM syndrome diagnosis, the identification of a specific TCM syndrome includes the main symptoms which refer to the common characteristics of the disease and the key clinical symptoms for the empirical formula. The secondary symptoms refer to the nontypical or individual symptoms for the disorders, which will provide important reference to herbal adjustments. Recipe means the empirical formula and herbal combination adjustment for the empirical basic formula. Main symptoms are the main index for choosing the empirical formula. Then the secondary symptoms are the complementary index for choosing the herbal adjustment strategy for the basic empirical formula. The objective of this procedure is to combine the two ways (i.e., empirical formula + main symptoms and herbal adjustment + secondary symptoms) to develop the final individualized herbal prescription for the patient. And in order to explore this procedure, we separately study Way 1 and Way 2 by the algorithm of association rules.

### 2.3. Analytic Procedure

**Figure 2:**
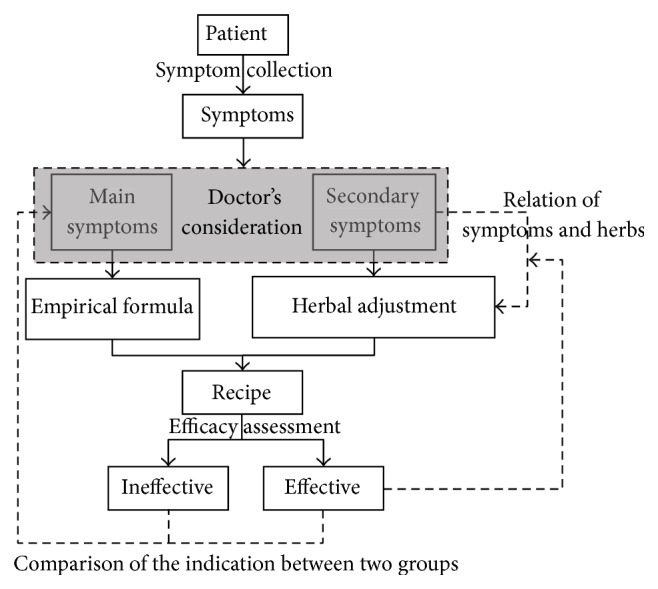
Flowchart of the study on pattern analysis of empirical herbal formula.

The analytic procedure for development patterns of the empirical herbal formula by TCM doctors is illustrated in Figure 2.

As indicated in Figure 2, the consideration patterns and procedure of a TCM doctor to develop an empirical formula are treated as a black box with implicit knowledge. The study is to visualize these patterns and procedure by the association rules method. The first process of choosing a basic empirical formula is to identify the main symptoms, in which the key indications for the empirical formula are to be detected with clear links to choosing. In clinical practice, TCM doctors make inevitable mistakes of inaccurately recognizing the indications and then choosing a wrong empirical formula. When the patients who are allocated to the ineffective group report ineffective outcome, they do have the indications of empirical formula. According to the comparison of symptoms between the invalid group and the effective group after the test, we analyzed the general indications of empirical formula and interpreted the relations between indications with association rules. And in the study of Way 2 (see Figure 1), the study objective is to find the relation between secondary symptoms and the herbal adjustment strategy based on the empirical herbal formula. According to the analytic result of Way 1, we cancelled the general indications among numerous symptoms. Then we found the individual symptoms for the patients. An association rule is built between the detected symptoms and herbal components as herbal adjustment strategy by the algorithm of association rules.

### 2.4. Inclusion and Exclusion Criteria for Patients

#### 2.4.1. Inclusion Criteria

The patients will be included in the study if they satisfy the following criteria:

1. the patient who is visited and treated by the empirical herbal formula of Professor Zhimin Yang for insomnia;
2. the patient who is diagnosed with insomnia in accordance with diagnosis of insomnia by the Chinese Mental Disorder Diagnosis III (CCMD-3) [5] and guidelines for clinical trials of new drugs of Traditional Chinese Medicine for treatment of insomnia [6];
3. the patient whose score is more than 7 by Pittsburgh Sleep Quality Index (PSQI);
4. the patient whose age ranges from 18 to 75;
5. the patient who is willing to participate and sign an informed consent document.

#### 2.4.2. Exclusion Criteria

1. Pregnancy or lactation women or those incapable of taking effective contraceptive measures in the study.
2. Patients incapable of understanding or communicating.
3. Patients who are participating in other clinical studies.
4. Patients with mental retardation, alcohol dependence, substance abuse, and suicide.
5. Patients with severe cardiovascular, cerebrovascular, lung, liver, kidney, and endocrine systems disease.
6. Patients with serious sleep syndrome.

### 2.5. Data Source and Study Period

All included patients are from the Neurology Clinic of Professor Zhimin Yang in Guangdong Provincial Hospital of Chinese Medicine. The study period is one year from January 2010 to January 2011.

The observed outcomes include demographic characteristics, medical history based on the four diagnostic methods of TCM, and assessed outcomes by the Pittsburgh Sleep Quality Index (PSQI). The conditions of patients are to be evaluated before the treatment and one month after the treatment.

### 2.6. Efficacy Evaluation and Analytic Strategy

The judgment standard of efficacy is as follows: PSQI reduced rate = [(PSQI of before-treatment − PSQI of post-treatment 1 month)/PSQI of before-treatment] × 100%.

It will be considered as totally recovered if the PSQI reduced rate > 75%, as excellently effective if the PSQI reduced rate ranges from 51% to 75%, as effective if the PSQI reduced rate ranges from 25% to 50%, and as ineffective if the PSQI reduced rate < 25%. The totally recovered, excellently effective, and effective cases are all allocated to the effective group and the ineffective cases make up the ineffective group.

The data were input by Epidata and analyzed by SPSS18.0 and Clementine12.0. The test significant level is *α* = 0.05 with two-sided Confidence interval.

### 2.7. Statistics

Record the data by Epidata. Analyze the data by SPSS18.0 chi-square and Clementine12.0. Association rules use GRI algorithm. Statistical test uses double-tailed test and *α* set 0.05.

## 3. Results

To the analytic procedure above, first, we should insure the effect of treatment, dividing the samples into an effective group and ineffective group. Second, compare symptoms between effective group and ineffective group and initially find the main symptoms. Third, use the association rule to determine the relationship between the main initial symptoms. Fourth, remove the main symptom and analyse the association of remaining symptoms and drug by association rule. Present the results as follows.

### 3.1. Demographic Characteristics

40 cases of confirmed insomnia patients from the Neurology Clinic of Professor Zhimin Yang are collected. All of them were treated by Professor Zhimin Yang with the empirical prescription of si-ni and gui-gang-long-mu Tang. The average age of the patients is 45.03 ± 12.17 years. 27.5% of them are male and 72.5% of them are female.

The longest medical history of insomnia ranges from 1 month to 240 months with the median of 120 months. 62.5% of the patients have midrange insomnia and 35.0% of them have serious insomnia.

### 3.2. Treatment Efficacy

The treatment efficacy is judged by PSQI reduction rate. The outcomes are classified into four levels: totally recovered, excellently effective, effective, and ineffective. The previous three levels (i.e., totally recovered, excellently effective, and effective) are allocated to the effective group and the last level is allocated to the ineffective group. We finally had 28 patients in the effective group and 12 patients in the ineffective group. The total effective rate was 70% (see Table 1).

### 3.3. Clinical Indication Filtering

206 features were investigated on each case. We filtered 11 features from the effective group and the invalid group by chi-square test (*P* < 0.05). Eight symptomatic patterns (i.e., fear of cold, fatigue, irascibility, palpitations, chilliness, ice cold limbs, thirst, and hidrosis) show positive correlation with the curative effect. Three symptomatic features (anxiety, constipation, and a red tongue tip) show negative correlation with the curative effect (see Table 2).

### 3.4. Relations among Clinical Indications

In the analysis of the clinical indications of the ineffective group, 10 patterns were identified with relations (frequency of “thirst” was zero) by the a priori algorithm based on association rules (support 10%, confidence 80%). Finally 4 rules were extracted from the ineffective group (see Table 3).

In the analysis of the clinical indications of the effective group, 11 patterns were identified with relations by the a priori algorithm (support 60%, confidence 80%). Finally 15 rules were extracted from the effective group (see Table 4).

### 3.5. The Herbal Adjustment Strategy

In the analysis of the herbal adjustment strategy for the empirical formula, all prescriptions were adjusted from the original formula based on the patients' condition. 26 kinds of herbal medicine were involved in the adjustment. For individual symptoms, we gathered 206 features in total and 11 common symptoms were excluded and 5 main symptoms of insomnia were cancelled during the analysis. 190 individual symptoms remained. In the analysis of the effective group, the GRI algorithm of association rules was used, in which the minimum support rate is 10%, the minimum confidence level is 90%, the amount of preitems is at most 3, and the rule is only to display the real. We are to analyze the association rules between items, which included 28 strategies of herbal adjustment, and the preitems which included 190 symptoms. 12 association rules are extracted, which include five kinds of herbal adjustment strategies (see Table 5).

## 4. Conclusion

### 4.1. Application of Association Rules to Empirical Formula

The algorithm based on association rules is capable of identifying the relations between sets of items in a large database. In the recent studies [7, 8], the association rules algorithms were applied to the empirical herbal arrangement of TCM. They were mainly used in drugs concerted application of retrospective medical records. However, it is seldom applied to the analysis of relations of clinical indication and empirical herbal prescriptions. Relevant studies on this area have not yet been reported.

In our study, we introduced a database for prospective observed cases in order to evaluate the efficacy of the herbal prescription after the patients were treated by the empirical herbal formula. Thus the study methodology is quite different from the retrospective analysis of medical records in the past. We believe this new study method can render a reliable conclusion for the efficacy of TCM empirical formula.

### 4.2. Indications of the Empirical Formula

The symptoms of anxiety, palpitations, and a red tongue tip show negative correlation with the efficacy of TCM empirical formula by univariate analysis. And the rules from the ineffective group had the symptom of palpitations. However, the rules from the effective group do not have the palpitations symptom. Therefore, the comparison among anxiety, a red tongue tip, and palpitations symptoms is important. And palpitations are an inhibition of the empirical prescription application.

In TCM, the rules extracted from the ineffective group (i.e., chilly→afraid of cold) and the effective groups (i.e., chilly→fatigue, ice cold limbs→afraid of cold) are regarded as the indication for the Yang or Qi asthenia syndrome. These rules exist both in the ineffective group and in the effective group. Therefore, the Yang or Qi asthenia syndrome is a necessary condition for the application of empirical herbal prescription. However, not all the patients who had Yang or Qi asthenia syndrome had effective outcomes to the empirical prescription. Therefore, the Yang or Qi asthenia syndrome is not a sufficient condition for the empirical prescription application.

Among the extracted rules from the effective group, 10 rules include the irascible symptom. These rules merge red the Yang or Qi asthenia syndrome but only exist in the effective group. Therefore, based on the Yang or Qi asthenia syndrome, we believe the irascible symptom is a sufficient condition of the empirical prescription application.

### 4.3. Herbal Adjustment Strategy for the Empirical Formula

In accordance with the TCM theory, the application of the empirical formula should be based on the main clinical symptoms or patterns of the patients. The herbal adjustment strategies for the empirical herbal formula depend on the symptoms of the individual patient. If we analyze all the collected clinical characteristics, it is not possible to discriminate whether the changes of herbal ingredients are the cause of temporary application ideas of empirical formula or a fix herbal adjustment strategy. Therefore, in this study we set 190 clinical features of disease excluding the indications of empirical formula and insomnia main symptoms as preitems. In that case, we can distinguish application ideas of the empirical formula and the drug of addition and subtraction. Because of rarely using some herbs, we only excavated 5 in a total of 28 kinds of addition and subtraction herbs.

### 4.4. Feedback by the Original Expert

Professor Yang gave her feedback to the above data analysis results. She considered that the indications of empirical prescription corresponded to the application thinking, and the 10th and 11th rules in addition and subtraction and clinical features are not her original application ideas of drugs of addition and subtraction. However, as a complement, they are important to further study in clinical practice. The remaining rules are consistent with her application ideas of herbal adjustment strategy.

## 5. Discussions

In summary, when compared with the conventional method based on statistics, the machine learning method of association rules has specific merits in analyzing the indications of empirical herbal prescription of TCM. And it is also good at exploring the relations among herbal adjustment for empirical formula. This method provides an effective and reliable approach for the study on TCM empirical herbal prescriptions in the future.

## Figures and Tables

**Table 1:** Treatment efficacy.

Efficacy judgment	Case	%
Ineffective	12	30.00
Effective	13	32.50
Excellently effective	14	35.00
Totally recovered	1	2.50

Total	40	100.00

**Table 2:** Related clinical indications.

Symptom	Group	Negative (%)	Positive (%)	χ^2^	*P*
Fear of cold	Ineffective	8 (66.7)	4 (33.3)	5.08	0.04
	Effective	8 (28.6)	20 (71.4)		
Fatigue	Ineffective	4 (33.3)	8 (66.7)	4.52	0.03
	Effective	2 (7.1)	26 (92.9)		
Anxiety	Ineffective	8 (66.7)	4 (33.3)	4.52	0.03
	Effective	26 (92.9)	2 (7.1)		
Irascibility	Ineffective	9 (75.0)	3 (25.0)	12.06	0.01
	Effective	5 (17.9)	23 (82.1)		
Palpitations	Ineffective	10 (83.3)	2 (16.7)	13.41	0.01
	Effective	6 (21.4)	22 (78.6)		
Chilliness	Ineffective	10 (83.3)	2 (16.7)	7.62	0.01
	Effective	10 (35.7)	18 (64.3)		
Ice cold limbs	Ineffective	11 (91.7)	1 (8.3)	9.31	0.01
	Effective	11 (39.3)	17 (60.7)		
Thirst	Ineffective	12 (100.0)	0 (0.0)	7.35	0.01
	Effective	16 (57.1)	12 (42.9)		
Hidrosis	Ineffective	11 (91.7)	1 (8.3)	6.22	0.03
	Effective	14 (50.0)	14 (50.0)		
Constipation	Ineffective	9 (75.0)	3 (25.0)	7.59	0.02
	Effective	28 (100.0)	0 (0.0)		
A red tongue tip	Ineffective	9 (75.0)	3 (25.0)	7.57	0.02
	Effective	28 (100.0)	0 (0.0)		

**Table 3:** Extracted rules from the ineffective group.

Consequent	Antecedent	Support (%)	Confidence (%)	Lift
Fatigue	Palpitations	16.67	100.00	1.50
Fear of cold	Chilliness	16.67	100.00	3.00
Fatigue	Constipation and fear of cold	16.67	100.00	1.50
Fear of cold	Constipation and fatigue	16.67	100.00	3.00

**Table 4:** Extracted rules from the effective group.

Consequent	Antecedent	Support (%)	Confidence (%)	Lift
Palpitations	Irascibility	82.14	86.96	1.11
Palpitations	Irascibility and fatigue	75.00	85.71	1.09
Fear of cold	Ice cold limbs	60.71	82.35	1.15
Fatigue	Ice cold limbs	60.71	94.12	1.01
Fatigue	Chilliness	64.29	100.00	1.08
Fatigue	Fear of cold	71.43	95.00	1.02
Fatigue	Palpitations	78.57	90.91	0.98
Fatigue	Irascibility	82.14	91.30	0.98
Fatigue	Palpitations and irascibility	71.43	90.00	0.97
Irascibility	Ice cold limbs	60.71	88.24	1.07
Irascibility	Chilliness	64.29	83.33	1.01
Irascibility	Palpitations	78.57	90.91	1.11
Irascibility	Fatigue	92.86	80.77	0.98
Irascibility	Chilliness and fatigue	64.29	83.33	1.01
Irascibility	Palpitations and fatigue	71.43	90.00	1.10

**Table 5:** Result of the association rules for relations between herbal adjustment strategy and clinical patterns in the effective group.

Preitems	Postitems	Support rate (%)	Confidence level (%)	Lift
Thirst and loose stools	White atractylodes rhizome	10.71	100.00	2.80
Thirst and loose stools	*Poria cocos *	10.71	100.00	2.33
Dizziness	*Poria cocos *	10.71	100.00	2.33
Dizziness and sense of suppression in the chest	*Poria cocos* with Rhizoma Pinellinae Praeparata	10.71	100.00	2.80
Greasy fur of tongue and short breath	*Poria cocos* with Rhizoma Pinellinae Praeparata	10.71	100.00	2.80
Greasy fur of tongue and pale complexion and short breath	*Poria cocos* with Rhizoma Pinellinae Praeparata	10.71	100.00	2.80
Postmenopausal women	Fresh dogwood with *Fructus mume *	17.86	100.00	1.56
Irritability	Fresh dogwood with *Fructus mume *	10.71	100.00	1.56
Sore throat	Fresh dogwood with *Fructus mume *	10.71	100.00	1.56
Tinnitus	Equivalent Guizhi with Aglaophotis radix herbaceu	10.71	100.00	2.00
Chest pain	Equivalent Guizhi with Aglaophotis radix herbaceu	10.71	100.00	2.00
Forgetfulness and breast pain	Equivalent Guizhi with Aglaophotis radix herbaceu	10.71	100.00	2.00
